# Single lateral approach for open reduction and internal fixation of posterior malleolar fragment in Weber B rotational ankle fracture

**DOI:** 10.1097/MD.0000000000032725

**Published:** 2023-01-20

**Authors:** Jaehyung Lee, Hwan Ryu, Jae Yong Park

**Affiliations:** a Department of Orthopaedic Surgery, Hallym University Sacred Heart Hospital, Hallym University College of Medicine, Anyang-si, Gyeonggi-do, Republic of Korea.

**Keywords:** ankle fractures, posterior malleolar (PM) fractures, surgical approach, trimalleolar fractures

## Abstract

Ankle fractures involving the posterior malleolus are a relatively common injuries, but various surgical approaches are still being introduced, and the selection of an appropriate surgical method is still controversial. The aim of this study was to introduce the surgical method using a single lateral approach for open reduction and internal fixation for posterior malleolar (PM) fractures associated with Weber B type ankle fractures. In this retrospective study, the single lateral approach was used for osteosynthesis of the PM fracture with Weber B lateral malleolar fractures. A total of 40 patients were followed up at for least 12 months (mean, 23.3; range, 12–88). Clinical assessment was based on the Olerud and Molander score, Foot and Ankle Outcome Score, visual analog scale, and subjective patient satisfaction 1 year after surgery. The accuracy of reduction was evaluated as <1 mm of displacement on the lateral view of the postoperative radiographs. The mean Olerud and Molander ankle score was 85.6 ± 12.7 and the mean Foot and Ankle Outcome Score was 82.7 ± 15.9 at 1-year postoperatively. Acceptable reduction was achieved in 38 of 40 (95%) cases. During the follow-up period, arthritic change was observed in 1 case and limited range of motion was confirmed in 2 cases. There was 1 case of postoperative wound problem and no case of sural nerve injury. The single lateral approach is a relatively simple and convenient method that enables accurate reduction and minimizing complication for fixation of the PM fractures with Weber B lateral malleolar fractures.

## 1. Introduction

Trimalleolar fracture is a relatively common form of trauma encountered in the orthopedic field. The presence of a posterior malleolar (PM) fracture increases joint instability, and often makes it more difficult to accurately reduce the articular surface.^[1–4]^ Among the fixation methods for PM fracture, anterior to posterior fixation is a technique of indirect reduction performed in supine position through a minimal incision, and has the advantages of short operating time and relative ease of procedure.^[5–7]^ However, depending on the fracture pattern, it can sometimes be difficult to reduce the fracture fragments, and when there are floating or impacted fragments within the joint it is virtually impossible to remove or manipulate these fragments.^[8,9]^

Due to these limitations, various authors have described methods of fracture site exposure and direct reduction via posteromedial or posterolateral approach.^[10–13]^ Unfortunately, most of these methods require the patient to be in a prone position, who then needs to switch positions during fixation of the accompanying medial malleolar fracture. Additionally, direct removal or manipulation of the incarcerated fragment often necessitates increased exposure and dissection, leading to injury of the surrounding soft tissue.

In this study, we introduce a surgical method for fixation of PM fracture accompanying Weber B lateral malleolar fracture using a single lateral approach performed in lateral position, and report on the results.

## 2. Methods

### 2.1. General information

The current study was approved by the institutional review board of Hallym University Sacred Heart Hospital (2021-03-002). The need to obtain informed consent was waived because of the retrospective nature of the study.

A retrospective review was performed on ankle fracture patients with Weber B lateral malleolar fracture and PM fracture who underwent surgery using a single lateral approach between March 2012 and December 2020 at our institution. Patients with other concomitant injuries, patients with history of previous ipsilateral ankle surgery, and patients aged <19 years were excluded. Only cases that were operated in lateral position with a single incision for both lateral malleolar and PM fractures were included, and cases that required conversion to prone position or additional incisions were excluded. Patients who were followed up for at least 12 months postoperatively were included, and the mean follow-up period was 23.3 (range, 12–88) months. A total of 40 patients were analyzed for this study.

**Figure 1.**
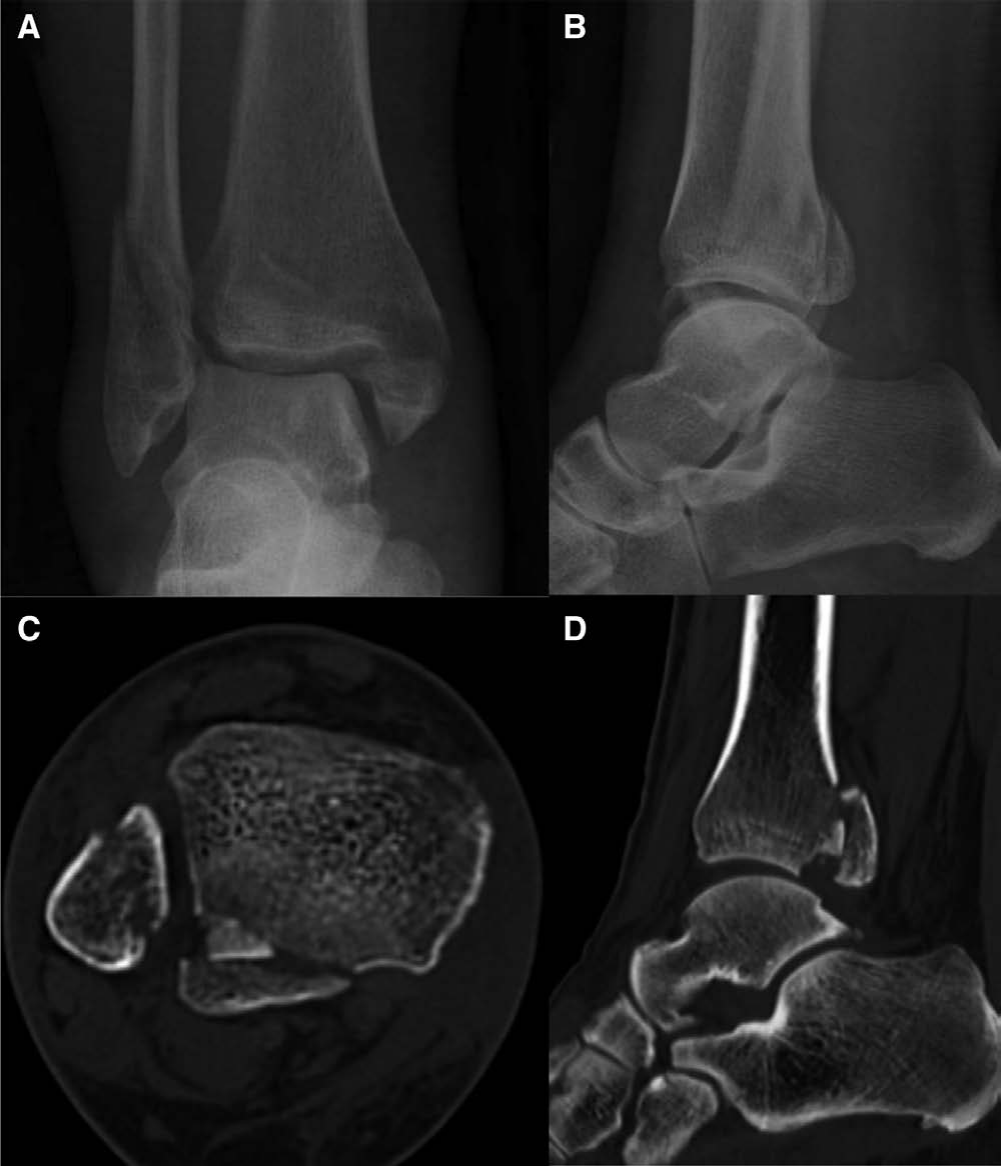
Pre-operative radiographs of the 52-year-old female patient with a trimalleolar fracture. (A) Anteroposterior and (B) lateral ankle plain radiographs. (C) Axial and (D) sagittal CT scans. An intra-articular incarcerated fragment was confirmed on CT. CT = computed tomography.

Plain radiographs and computed tomography (CT) scans were used to determine PM fracture pattern. Preoperative lateral ankle radiographs were evaluated to see whether the fracture extended to >25% of the articular surface. There were 27 cases of the fracture involving >25% of the articular surface, and 13 cases involving <25%. Based on the CT scans, PM fracture patterns were divided into type 1, 2, and 3 according to the Haraguchi classification.^[14]^ Fifteen cases were classified as type 1, 18 as type 2, and 7 as type 3. The presence and size of intra-articular incarcerated fragments were confirmed by CT (Fig. 1). The size of the incarcerated fragment was >3 mm in 16 cases, <3 mm in 13 cases, and no fragment in 11 cases.

Indications for surgery and whether to use the single lateral approach was decided by the senior author (JYP) after careful consideration of articular incongruity, syndesmotic instability, intra-articular step-off and the presence of incarcerated fragments.

### 2.2. Clinical analysis

Olerud and Molander score, Foot and Ankle Outcome Score, visual analog scale (VAS), and subjective patient satisfaction were recorded 1 year postoperatively. Olerud and Molander score is a 9-item questionnaire to assess patient reported outcome following a fracture of the ankle.^[15]^ It contains single response, multiple choice questions and scores from 0 to 100, with higher scores indicating better outcomes.^[16]^ Foot and Ankle Outcome Score consists of 5 domains and 100 points total (pain, other symptoms, function in activities of daily living, function in sport and recreation, and foot and ankle related quality of life), with 100 points indicating the best results.^[17]^ The 10-mm VAS is a validated self-assessment tool for evaluating pain. Patients were asked to categorize their current state as “very satisfied,” “satisfied,” “fair,” “dissatisfied,” or “very dissatisfied.” They were also asked 2 questions: “If you had a fracture in the opposite ankle, would you have the same treatment again?,” and “If your family member or close friend sustained the same injury, would you recommend getting the similar treatment as yours?” Patients could answer: likely, neither likely nor unlikely, unlikely, or don’t know.^[18]^ In addition, operative time and presence of postoperative complications during follow-up were also investigated. Potential complications included wound problems, arthritis, sural nerve injury, and limited range of motion (ROM). ROM limitation was defined as a limitation of >10 degrees of motion compared to the unaffected side.

### 2.3. Operative technique

**Figure 2.**
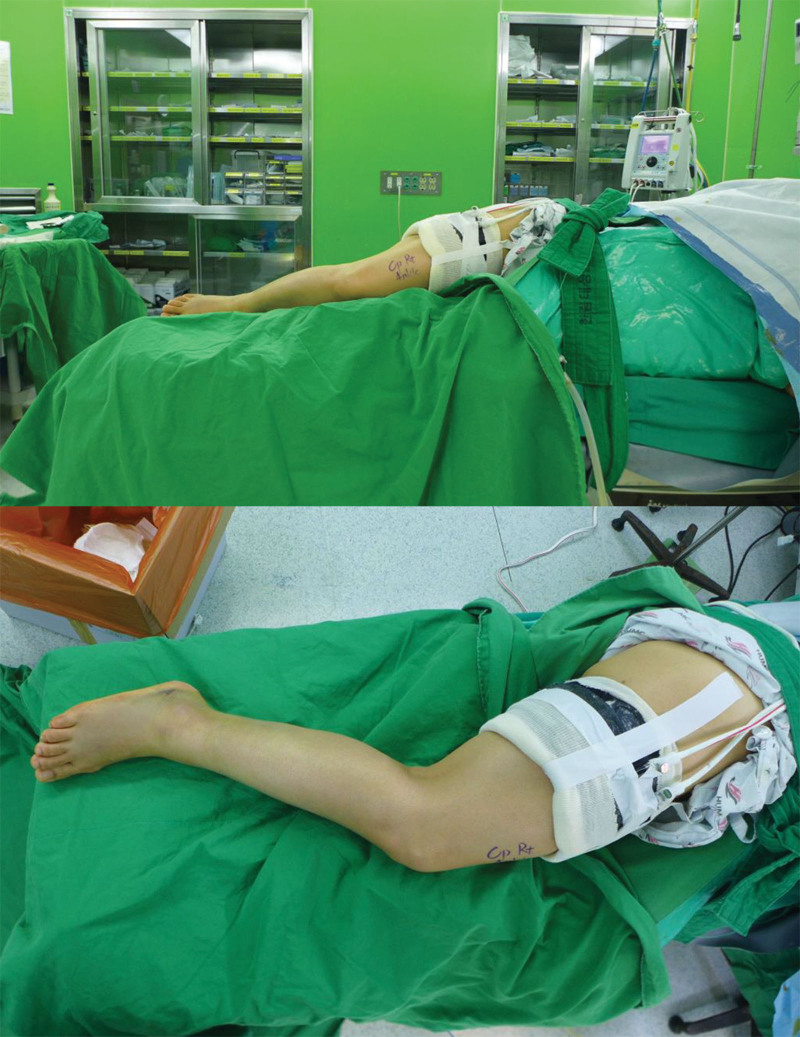
With a pneumatic tourniquet applied, the patient was placed in a lateral position on a vacuum bean bag positioner.

**Figure 3.**
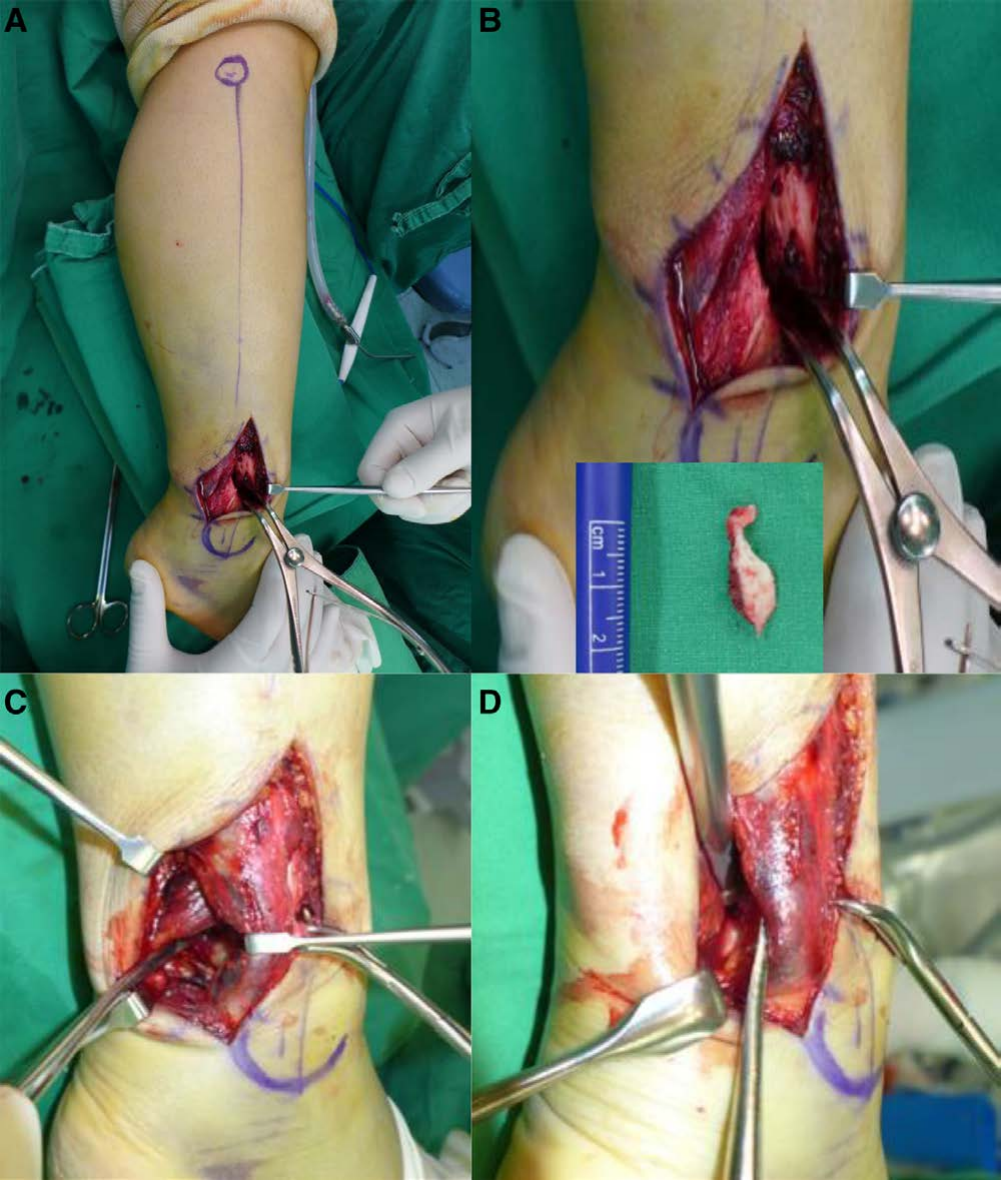
Intraoperative findings. (A) The fibula fracture site was spread using a lamina spreader. (B) The posterior malleolar fracture site was approached through the gap between the fibula fragments, and the bony fragment stuck in the joint was removed. (C) After fixation of the fibular fracture with a lag screw, dissection was performed between the peroneal muscle and the flexor hallucis longus muscle. (D) The posterior malleolar fragment was pressed with an instrument such as a ball spike pusher to maintain reduction.

**Figure 4.**
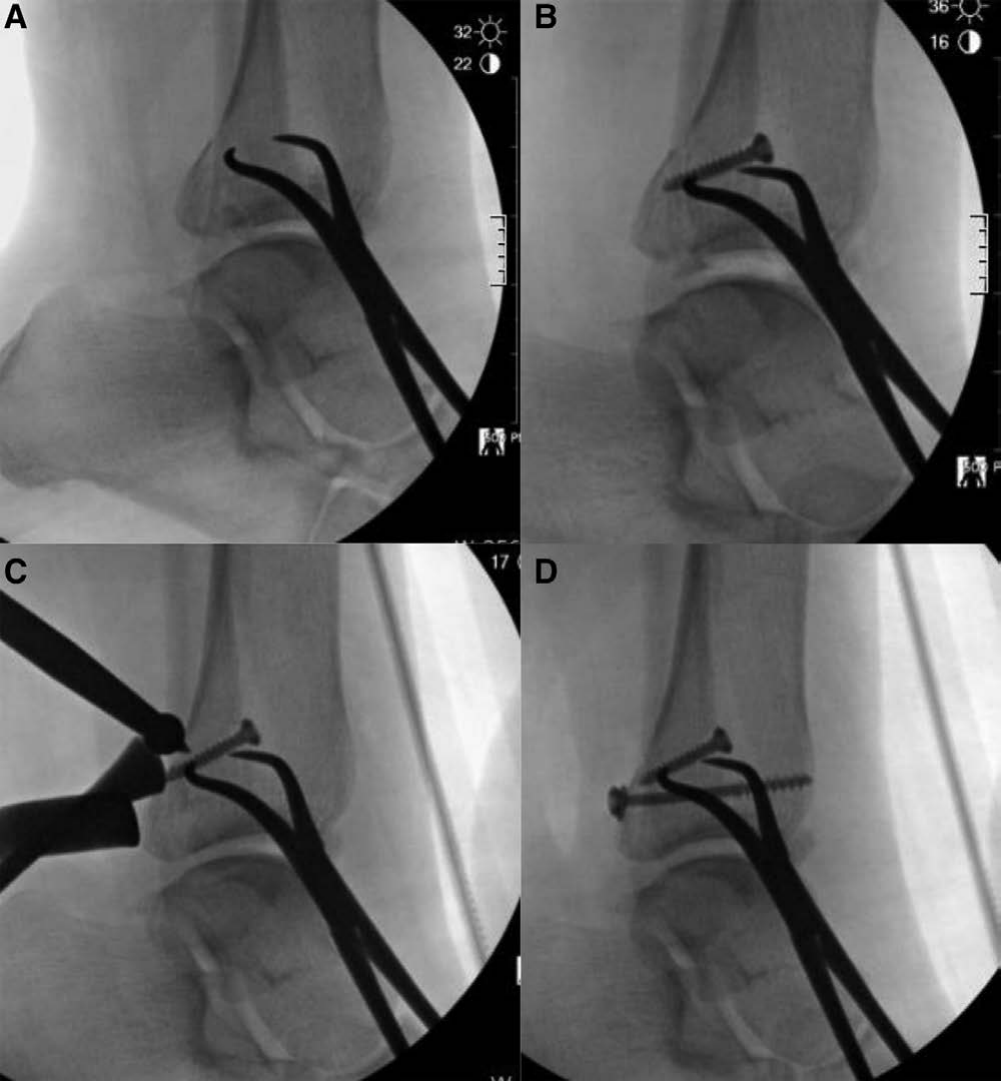
Intraoperative C-arm images. (A) The fibula fracture site was reduced using a reduction clamp, (B) and the fibula was fixed with a lag screw. (C) While maintaining reduction by pushing the posterior malleolar fragment toward the articular surface with a ball spike pusher, (D) the fragment was fixed in the posterior to anterior direction with a partially threaded screw.

With the tourniquet applied, the patient was placed in a lateral position on a vacuum bean bag positioner (Fig. 2). A longitudinal incision or longitudinal-curved incision was made along the center of fibula to expose the fibular fracture site. Distraction of the fibular fracture site using a lamina spreader allowed access to the PM fracture plane (Fig. 3A). To prevent damage to intact portion of the interosseous membrane, the fibula fracture site should be carefully spread apart. It enabled easy visualization of the PM fracture site and posterior talar dome.^[19]^ And then, the hematoma between the distal tibia and the PM fracture fragment was removed. In the case with an intra-articular incarcerated bony fragment, the fragment was reduced by pushing to the anterior distal tibia base and the inferior talar dome. If the incarcerated fragment was too small or irreducible, it was removed (Fig. 3B). Afterwards, the fibular fracture was reduced and fixed with a lag screw. In some cases, when the lag screw alone was not sufficient to ensure a firm fixation, reduction was maintained using a reduction clamp while the remainder of surgery for the PM fracture was performed. Since the hematoma interposed in the PM fracture plane was initially removed, reduction of the fibular at this point also reduces the PM fragment in most cases due to the ligamentotaxis effect of the posterior inferior tibiofibular ligament. For fixation of the PM fragment, an approach between the peroneal muscles and the flexor hallucis longus muscle allows exposure to the posterior surface of the PM fragment (Fig. 3C). After obtaining a true lateral view of the ankle joint using a C-arm, the fragment was pressed with an instrument such as a ball spike pusher to maintain reduction, and fixation was performed using a partially threaded cancellous screw near the physeal scar level (Figs. 3D and 4). One screw was used in 29 cases, 2 screws were used in 10 cases, and 1 screw with 1 Kirschner wire were used in 1 case. Then the locking compression fibular plate (DePuy Synthes, Warsaw, IN) was applied. Two proximal locking screws and 3 distal locking screws were used for fixation of the fibula. The reason for applying the fibula plate after PM fragment fixation is that it is difficult to visualize the PM fracture site and tibiotalar joint surface in the ankle lateral view with the plate already fixed on the fibula. Additionally, in some cases with anterior inferior tibiofibular ligament (AITFL) injury or AITFL avulsion fracture, suture or fixation were performed using a suture anchor or a screw. If additional fixation for medial malleolar fracture was necessary after the procedure for the lateral malleolar was completed, the patient was switched to supine position while maintaining a sterile drape, and the procedure for medial malleolar fracture was carried out.

### 2.4. Postoperative care

After surgery, careful consideration of the patient’s bone quality, soft tissue condition, fracture pattern, patient’s general condition and compliance factored into determining the rehabilitation and weight-bearing regimen. In general, ROM exercises and partial weight-bearing were started with splints or braces applied 2 weeks after surgery. From 6 weeks after surgery, full-weight bearing was allowed. In instances of poor bone quality or severe injury, non-weight bearing was maintained until 4 to 6 weeks after surgery, and then gradually allowed into weight-bearing.

## 3. Results

**Figure 5.**
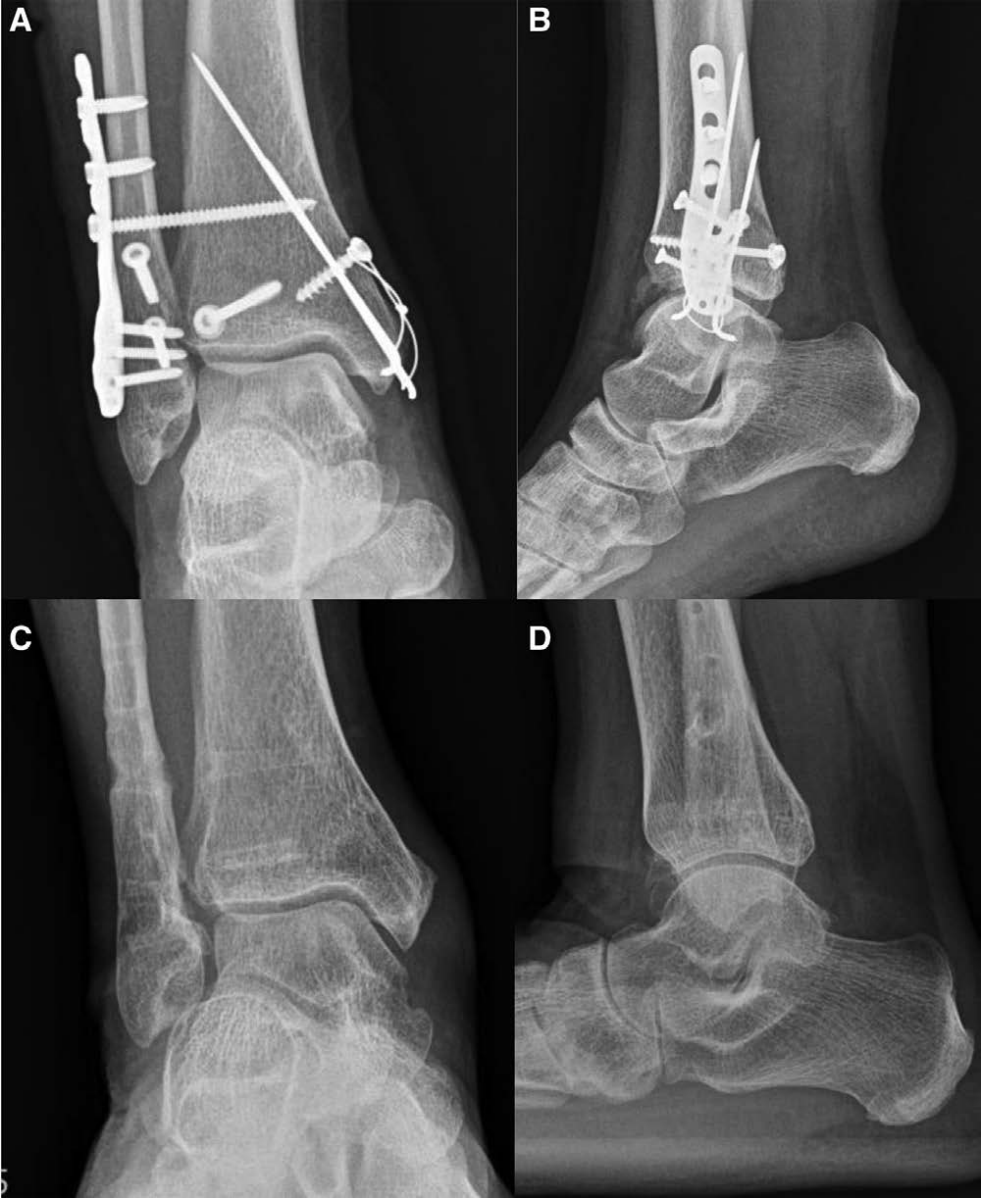
Postoperative image of the patient presented in Figure 1. Immediate postoperative (A) anteroposterior and (B) lateral ankle plain radiographs. Postoperative 1-year (C) anteroposterior and (D) lateral ankle plain radiographs. The articular surfaces are well preserved and there is no evidence of post-traumatic arthritis.

Patient characteristics and descriptive statistics are presented in Table 1. Accurate reduction was achieved in 38 of 40 PM fractures (<1 mm of displacement in the lateral view of the postoperative radiographs) (Fig. 5). The Olerud and Molander ankle score, foot and ankle outcome score, and VAS at 1 year after surgery showed good results (Table 2).

**Table 1:** Demographics and descriptive values.

Variable		Value	
Age (yr)		52.5	22–82
Gender	Male	12	30%
	Female	28	70%
Danis–Weber classification	Type B	40	100%
Fracture type	Trimalleolar	30	75%
	Trimalleolar & dislocation	7	17%
	Trimalleolar equivalent	3	8%
Involvement of tibial articular surface of the posterior malleolar fracture*	<25%	13	32.00%
	>25%	27	68.00%
Intra-articular incarcerated fragment†	>3mm	16	40%
	<3mm	13	32%
	None	11	28%
Haraguchi classification	Posterolateral oblique (type I)	15	38%
	Medial extension (type II)	18	45%
	Small shell (type III)	7	17%
Time interval from trauma to surgery (d)		2.7	0 to 7
Anesthesia	General	21	53%
	Spinal	19	47%
Posterior malleolar fixation	1 screw	29	73%
	2 screws	10	25%
	1 screw + 1 Kirschner wire	1	2%
Operative time (min)	Trimalleolar	87	62–115
	Trimalleolar & dislocation	96	70–131
	Trimalleolar equivalent	57	45–85
Follow-up duration (mo)		23.3	12–88

The values are presented as mean (range) or n (%).

* Measured on preoperative lateral radiographs.

† Measured on preoperative computed tomography (CT) scans.

**Table 2:** Clinical scores at 1 year postoperatively.

Variable	Value
Olerud and Molander ankle score (100)	85.6 ± 12.7
Foot and Ankle Outcome Score (100)	82.7 ± 15.9
VAS (10)	1.6 ± 1.5

The values are presented as mean ± standard deviation.

VAS = visual analog scale.

Twenty-five patients indicated that they were satisfied with the surgical results, and 2 patients responded as dissatisfied (Table 3). When asked whether they would receive the same treatment if their contralateral ankle sustained an identical injury, 22 responded yes and 1 answered no. When asked if they would recommend this treatment to others, 21 responded likely, and 2 answered unlikely. The rest either answered that they did not know or refused to respond (Tables 4 and 5).

**Table 3:** Patient subjective satisfaction at 1 year postoperatively.

Response	n = 40
Very satisfied	14
Satisfied	11
Fair	5
Dissatisfied	2
Very dissatisfied	0
No response	8

**Table 4:** If you had a fracture in the opposite ankle, would you have the same treatment again?.

Response	n = 40
Likely	22
Neither likely nor unlikely	3
Unlikely	1
Don’t know	6
No response	8

**Table 5:** If your family member or close friend sustained the same injury, would you recommend getting the similar treatment as yours?.

Response	n = 40
Likely	21
Neither likely nor unlikely	3
Unlikely	2
Don’t know	6
No response	8

Complications included 1 case of postoperative wound problem, where bullae formation occurred around the surgical site, which recovered after routine wound dressing and did not require wound revision surgery. At 1 year after surgery, arthritic change was observed in 1 case and ROM limitation was confirmed in 2 cases. There were no cases of sural nerve injury (Table 6).

**Table 6:** Complications after surgery.

Complications	n = 40
Wound problem	1
Arthritic change	1
ROM limitation*	2
Sural nerve injury	0

ROM = range of motion.

* ROM limitation was defined as a case in which a dorsiflexion or plantarflexion range decreased by >10 degrees compared to the unaffected side.

## 4. Discussion

Concomitant PM fracture in cases of ankle fracture creates a more unstable joint compared to cases without PM fracture, and it is often difficult to achieve accurate reduction of the joint surface. For these reasons, the prognosis for trimalleolar fractures have been reported to be poorer than bimalleolar fractures.^[4,20,21]^ As it became known that a large posterior fracture fragment has a poor prognosis if treated nonoperatively, the lateral view radiograph of the ankle joint has long been used to determine whether the fracture involves 25% or 33% of the articular surface and thus requires fixation.^[1,6,12,13,22]^ However, due to the proliferation of 3D CT analysis, other criteria are increasingly being emphasized. Blom et al recently reported that the clinical prognosis after PM fracture treatment was related to the type of fracture and remaining intra-articular step-off after treatment, but was not associated with the size of the fracture fragment.^[23]^ Various authors have reported and suggested that in addition to the size of the PM fracture fragment, other factors such as syndesmosis instability, incarcerated small fragment in the articular surface between the fracture, and fracture pattern extending medially or to the fibular notch were also important in determining the surgical treatment and prognosis.^[11,22,24–26]^

Among PM fracture fixation methods, anterior to posterior screw fixation is a technique that has long been used and adapted widely.^[6,26,27]^ It has the advantages of short operating time and relatively simple technique, but it can only be applied when the PM fragment is large enough, and depending on the fracture pattern it can be difficult to reduce the fragment. In addition, it is virtually impossible to remove or manipulate any floating fragments or incarcerated fragments within the joint. Numerous studies have reported that the incidence of postoperative articular step-off was significantly higher in anterior to posterior screw fixation than posterolateral fixation, and the clinical outcomes were also reported to be superior when using the latter method.^[7,12,28,29]^

The direct reduction and fixation method of PM fracture through a posterolateral approach has the advantages of direct visualization of the fracture plane, accessible manipulation of the fragment, and accurate restoration of the articular surface.^[11,22,26,30]^ This method is usually performed with the patient in a prone position, which offers an unobstructed view of the fracture site and makes it easy to apply either screws or plates depending on the fracture type. However, in cases of trimalleolar fracture, it may be a little unfamiliar to perform reduction and fixation for a medial malleolar fracture in the prone position, and in the case of a lateral malleolar fracture with accompanying chaput fragment, manipulation of the chaput fragment or AITFL rupture is almost impossible in prone position. Converting the patient to supine position in such cases requires additional draping and extended operating time.

In this study, we introduced a surgical technique using a single lateral approach in the lateral position during fixation for PM fracture accompanying Weber B type lateral malleolar fracture, and the results were reported. By approaching the PM fracture site through a transfibular approach, removal of the hematoma and manipulation of the incarcerated fragment was simple and straightforward. Also, the subsequent approach between the peroneal muscles and the flexor hallucis longus muscle to perform screw fixation could be done without any additional skin incisions, thus minimizing further tissue dissection whilst allowing for accurate reduction and shortening operating time. In addition, since it is performed in a lateral rather than prone position, it is less cumbersome to change the patient to supine position without the need for aseptic re-draping when surgery for medial malleolar fracture is necessary. This surgical method allows manipulation of incarcerated fragments, which cannot be performed in the anterior to posterior screw fixation technique, and has the advantage that reduction and fixation are possible through less manipulation of surrounding tissues in the lateral position, unlike the traditional posterolateral approach. However, since manipulation of the PM fragment may not be as easy as compared to the posterolateral approach, the ligamentotaxis effect of the posterior inferior tibiofibular ligament that occurs simultaneously with the fibular fixation during the reduction of the PM fragment is important. If the time of surgery is too delayed from the time of injury, there may be limitations in receiving help from this ligamentotaxis effect. For this reason, in this study, surgery was performed within an average of 2.7 days (range, 0–7) from the time of injury.

In this study, radiographically accurate reduction (<1 mm of displacement in the lateral radiographs) was confirmed in 38 of 40 cases. This is an equal or superior result compared to studies using other surgical methods.^[10,31]^ In addition, compared to the surgical methods introduced in other studies performed in the prone position, our study has the advantage of being able to obtain accurate reduction of the PM with a simpler method in the lateral position.

Clinical scores and subjective patient satisfaction of patients were also good, and there were no severe complications such as a sural nerve injury or wound problems requiring surgical debridement. Sural nerve injury is a complication known to occasionally occur when using the posterolateral approach, and Jowett et al reported that when the incision is made midway between the Achilles tendon and the lateral malleolus, the sural nerve can cross the incision line at 56.7 mm proximal to the tip of the lateral malleolus.^[32]^ Solomon et al discovered that the sural nerve was on average 7 mm posterior to the most prominent edge of the lateral malleolus and on average 13 mm distal to the tip.^[33]^ During surgery, we made an incision along the center of the fibula, minimized dissection at the subcutaneous level, and retracted the flexor hallucis longus posteromedially to protect the sural nerve. We assume this technique contributed to preventing neural complications. However, since there may be variations in the course of the sural nerve, utmost care is needed even when using the approach presented in this study.^[34]^

This study has several limitations. First, it was performed as a retrospective study, and because the indications for surgery and selection of approach were decided according to the surgeon’s judgment, there may be a personal bias. In addition, due to the relatively small sample size, potential complications may not have all been properly identified. Also, since this study did not compare the results with a traditional posterolateral approach or other approaches, it cannot definitively report superior outcomes compared to other methods. Further comparative or prospective studies with larger numbers of patients may help to evaluate the effectiveness of this approach.

In conclusion, it is increasingly emphasized that accurate reduction and fixation of the PM fragment plays an important role in securing the stability of the ankle joint including syndesmosis. In this study, it was found that the single lateral approach is a relatively simple and convenient method that enables accurate reduction and minimizing complication. We recommend using this surgical technique for fixation of the PM fractures with Weber B lateral malleolar fractures.

## Author contributions

**Conceptualization:** Jaehyung Lee, Jae Yong Park.

**Data curation:** Jaehyung Lee, Hwan Ryu.

**Formal analysis:** Jaehyung Lee, Hwan Ryu.

**Funding acquisition:** Jae Yong Park.

**Investigation:** Jaehyung Lee, Hwan Ryu.

**Methodology:** Jaehyung Lee, Jae Yong Park.

**Project administration:** Jaehyung Lee.

**Resources:** Hwan Ryu.

**Supervision:** Jaehyung Lee, Jae Yong Park.

**Validation:** Jaehyung Lee, Hwan Ryu.

**Visualization:** Hwan Ryu.

**Writing – original draft:** Jaehyung Lee, Hwan Ryu.

**Writing – review & editing:** Jaehyung Lee, Jae Yong Park.
